# Machine learning explainability in nasopharyngeal cancer survival using LIME and SHAP

**DOI:** 10.1038/s41598-023-35795-0

**Published:** 2023-06-02

**Authors:** Rasheed Omobolaji Alabi, Mohammed Elmusrati, Ilmo Leivo, Alhadi Almangush, Antti A. Mäkitie

**Affiliations:** 1 Research Program in Systems Oncology, Faculty of Medicine, University of Helsinki, Helsinki, Finland; 2 Department of Industrial Digitalization, School of Technology and Innovations, University of Vaasa, Vaasa, Finland; 3 Institute of Biomedicine, Pathology, University of Turku, Turku, Finland; 4 Department of Pathology, University of Helsinki, Helsinki, Finland; 5 Faculty of Dentistry, Misurata University, Misurata, Libya; 6 Department of Otorhinolaryngology-Head and Neck Surgery, University of Helsinki and Helsinki University Hospital, Helsinki, Finland; 7 Division of Ear, Nose and Throat Diseases, Department of Clinical Sciences, Intervention and Technology, Karolinska Institute and Karolinska University Hospital, Stockholm, Sweden

**Keywords:** Computational biology and bioinformatics, Health care, Medical research, Oncology, Cancer, Computational science, Computer science, Information technology

## Abstract

Nasopharyngeal cancer (NPC) has a unique histopathology compared with other head and neck cancers. Individual NPC patients may attain different outcomes. This study aims to build a prognostic system by combining a highly accurate machine learning model (ML) model with explainable artificial intelligence to stratify NPC patients into low and high chance of survival groups. Explainability is provided using Local Interpretable Model Agnostic Explanations (LIME) and SHapley Additive exPlanations (SHAP) techniques. A total of 1094 NPC patients were retrieved from the Surveillance, Epidemiology, and End Results (SEER) database for model training and internal validation. We combined five different ML algorithms to form a uniquely stacked algorithm. The predictive performance of the stacked algorithm was compared with a state-of-the-art algorithm—extreme gradient boosting (XGBoost) to stratify the NPC patients into chance of survival groups. We validated our model with temporal validation (n = 547) and geographic external validation (Helsinki University Hospital NPC cohort, n = 60). The developed stacked predictive ML model showed an accuracy of 85.9% while the XGBoost had 84.5% after the training and testing phases. This demonstrated that both XGBoost and the stacked model showed comparable performance. External geographic validation of XGBoost model showed a c-index of 0.74, accuracy of 76.7%, and area under curve of 0.76. The SHAP technique revealed that age of the patient at diagnosis, T-stage, ethnicity, M-stage, marital status, and grade were among the prominent input variables in decreasing order of significance for the overall survival of NPC patients. LIME showed the degree of reliability of the prediction made by the model. In addition, both techniques showed how each feature contributed to the prediction made by the model. LIME and SHAP techniques provided personalized protective and risk factors for each NPC patient and unraveled some novel non-linear relationships between input features and survival chance. The examined ML approach showed the ability to predict the chance of overall survival of NPC patients. This is important for effective treatment planning care and informed clinical decisions. To enhance outcome results, including survival in NPC, ML may aid in planning individualized therapy for this patient population.

## Introduction

Nasopharyngeal carcinoma (NPC) is an uncommon cancer showing distinctive epidemiology and histopathology which is different from other head and neck cancers^1–3^. It is endemic in the Southern China and Southeast Asia geographic locations where a significant amount of the tumors are undifferentiated and nonkeratinizing carcinomas^4–6^. But in nonendemic geographic locations, NPC can be either keratinizing or nonkeratinizing^6,7^. Notably, NPC initiates from the epithelial lining of the nasopharynx and thus the upper part of the pharynx^8^.

Recently, NPC has received significant attention as a global health concern due to its significantly increased incidence and mortality rates^9^. Additionally, regardless of early diagnosis, the mortality rate of NPC is considerably high irrespective of the geographic location—endemic or non-endemic^10^. This may be due to improper treatment planning producing suboptimal treatment outcomes^11^. Therefore, accurate estimation of the prognosis of NPC patients is important for effective management of the disease as the increase in the number of cancer patients with poor prognoses will increase the overall cancer burden in the society^1,12^.

The tumor-nodal-metastasis (TNM) staging scheme remains the cornerstone of prognostication and risk stratification for NPC patients^1^. Nevertheless, there are growing criticisms about TNM staging, as patients at the same stage may show significant clinical heterogeneity and unique oncologic outcomes^13^. Similarly, plasma Epstein-Barr Virus (EBV) DNA titer has been reported to be a useful biomarker for patients with NPC^14,15^. However, the financial and economic implications of examining EBV DNA and interlaboratory variability constitute significant factors hindering the integration of this biomarker in daily clinical practice^16^. Therefore, there is an ongoing discussion regarding the incorporation of non-anatomical prognostic factors that would reflect biological tumor behavior in addition to the TNM parameters for improved risk stratification^8,17^. An insightful potential approach to considering other factors in addition to the TNM staging scheme is the use of machine learning (ML), a subfield of artificial intelligence^18,19^.

Several studies have examined the use of various individual ML algorithms in the prognostication of outcomes in NPC^18,20^. In this study, we aim to leverage the performance of five different individual algorithms—logistic regression, naïve Bayes, k-nearest neighbors, support vector machine, and decision tree algorithms to produce a single distinct ML algorithm known as a stacked algorithm (stacking generalization). Additionally, we aim to compare the performance of the stacked algorithm with another state-of-the-art algorithm called extreme gradient boosting (XGBoost) ML algorithms for the prognostication of overall survival outcomes in NPC cancer patients. Extreme gradient boosting was chosen because it has achieved promising results in many clinical applications^21^. We provided an explanation and interpretation of the predictions made by the XGBoost model using the Local Interpretable Model Agnostic Explanations (LIME) and SHapley Additive exPlanations (SHAP) techniques. The resulting explainable and interpretable model may aid in prognostication by assisting in personalized chance of survival stratification for the patients; thus, adequate treatment intensity can be tailored for the patient.

## Material and methods

### Dataset

Approval was obtained from the National Cancer Institute (NCI) database through the Surveillance, Epidemiology, and End Results (SEER) Program of the National Institutes of Health (NIH) database with identification number (#17247-Nov2020 [alabir]/SAR0058552 [2023]). Written informed consent was obtained for all participants through electronic research administration (eRA) for SEER and NIH. This publicly available database was selected because it contains high-quality cases of various cancers in a non-identifiable format^22,23^. All methods were carried out in accordance with Helsinki declaration. In addition, all methods used in this study followed the SEER guidelines.

### Selection of patient attributes

**Figure 1:**
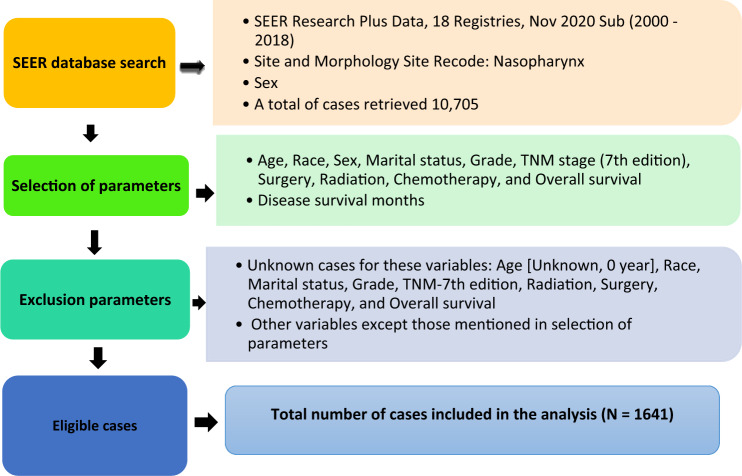
Extraction process from the SEER database.

The SEER program of the National Cancer Institute database was searched for Nov 2020 submission [2000–2018] (Fig. 1). The selected clinicopathological variables for nasopharyngeal carcinoma were the American Joint Committee on Cancer (AJCC) tumor-nodal-metastasis (TNM) 7th edition staging scheme, age at diagnosis, race, marital status, gender, and grade. The treatment-related parameters included surgery, radiotherapy (RT), chemoradiotherapy (CRT), and chemotherapy (CT) (Table 1). The survival period (in months) and overall survival status of the patients were also recorded.

**Table 1:** Baseline demographic and tumor characteristics of nasopharyngeal cancer patients in the SEER database.

Variables (definition)	Total (N = 1094) (%)	Categorization for ML analysis	Data type after categorization
Race			
Ethnicity of the patient			
White	462 (42.2%)	0 = White	Numeric
Black	89 (8.1%)	1 = Black	
Others	543 (49.6%)	2 = Others (American Indian/AK Native, Asian pacific)	
Age at diagnosis			
Age of the patient at diagnosis			
< 40 years old (young)	169 (15.5%)	No categorization	Discrete
> = 40 years old (old)	925 (84.5%)		
Gender			
Biological sex			
Female	338 (30.9%)	0 = Female	Numeric
Male	756 (69.1%)	1 = Male	
Marital status			
Marital status of the patient at the time of diagnosis of NPC			
Single (never married)	397 (36.3%)	0 = Single (never married)	Numeric
Married	697 (63.7%)	1 = Married	
AJCC 7th edition, T-stage (2010–2015)			
AJCC T1	393 (35.9%)	T1 = 1	Numeric
AJCC T2	206 (18.8%)	T2 = 2	
AJCC T3	222 (20.3%)	T3 = 3	
AJCC T4	273 (25.0%)	T4 = 4	
AJCC 7th edition, N-stage (2010–2015)			
AJCC N0; No regional lymph node metastasis	342 (31.3%)	N0 = 0	Numeric
AJCC N1; Single node regional lymph node metastasis	389 (35.6%)	N1 = 1	
AJCC N2; Cancer has spread to single lymph nodes	361 (33.0%)	N2 = 2	
AJCC N3; Cancer has spread to one or more lymph node	2 (0.2%)	N3 = 3	
AJCC 7th edition, M-stage (2010–2015)			
AJCC M0; No distant metastasis	1001 (91.5%)	M0 = 0	Numeric
AJCC M1; Presence of distant metastasis	93 (8.5%)	M1 = 1	
Grade			
The differentiation of cancer cell			
Grade I: Well differentiated	34 (3.1%)	Grade I = 1	Numeric
Grade II: Moderately differentiated	148 (13.5%)	Grade II = 2	
Grade III: Poorly differentiated	440 (40.2%)	Grade III = 3	
Grade IV: Undifferentiated	472 (43.1%)	Grade IV = 4	
Surgical resection			
Indication of the performance of surgery			
No surgery performed	935 (85.5%)	0 = No surgery performed	Numeric
Surgery performed	159 (14.5%)	1 = Surgery performed	
Radiotherapy			
This describes whether the patient receives radiation or not			
Exposure to radiotherapy	159 (14.5%)	1 = Exposure to radiation	Numeric
No exposure to radiation therapy	935 (85.5%)	0 = No exposure to radiation	
Chemotherapy			
No chemotherapy administered	203 (18.6%)	0 = No chemotherapy administered	Numeric
Chemotherapy was administered	891 (81.4%)	1 = Chemotherapy was administered	
Overall status			
Alive	658 (60.1%)	0 = Alive	Numeric
Dead	436 (39.9%)	1 = Dead	

From this extraction process (Fig. 1), a total of 1641 cases were found to be eligible for inclusion in this study (Table 1). Out of these 1641, a total of 1094 cases were used in the ML analysis for model training and internal validation.

Due to the rarity of NPC and the consequent lack of publicly available data, the remaining 547 cases were neither used for training nor testing during the model training or internal validation but reserved for a temporal form external validation of the developed model ("External validation, performance metrics, and feature importance"). The temporal form of external validation was emphasized by Ramspek et al., especially in the absence of a relatively large independent geographic external validation cohort^24,25^. The detailed description of each of the included variables and categorizations is shown in Table 1. All unknown cases were excluded.

### Machine learning model training

**Figure 2:**
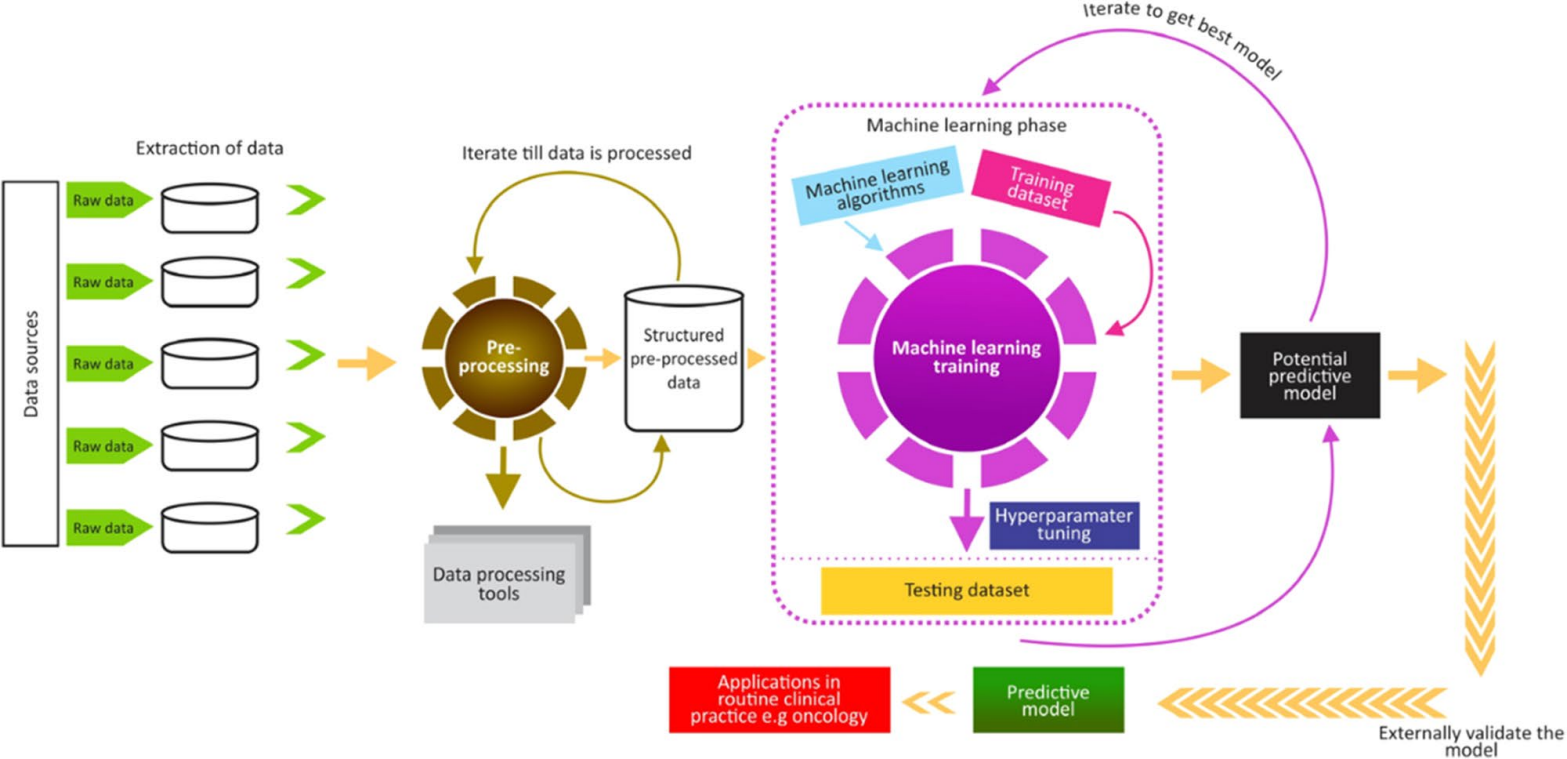
A typical ML training process.

A detailed description of the ML process is presented in Fig. 2. The process begins with data processing, where the data are converted into numeric variables for an easy ML process. The processed data were divided into input and output parameters.

From the parameters presented in Table 1, overall survival (OS) was considered the output of interest in this study. The output variable is relatively balanced, hence, there was no concern of a significantly imbalanced dataset. To obtain the staked algorithm, each of the five algorithms (logistic regression, naïve Bayes, k-nearest neighbors, support vector machine, and decision tree algorithms) were trained using tenfold cross-validation. Additionally, the necessary hyper-parameters were tuned to ensure that promising predictive performance was achieved. Then, all these algorithms were staked together with logistic regression as the base algorithm. The resulting staked algorithm was further re-trained on the entire dataset. The performance of the individual algorithms and the stacked algorithm were compared.

Similarly, we used the same data to train another state-of-the-art algorithm—extreme gradient boosting (XGBoost). Hyperparameters were fine-tuned to maximize the performance of the model (Fig. 2). Furthermore, we compared the performance of these two powerful algorithms (stacked and XGBoost) mainly based on accuracy. The algorithm with better performance accuracy was subjected to external validation. The ML training was done using Python version 3.11.0 in Jupyter notebook. The trained model was used to stratify the patients into two groups in terms of the chance of OS, i.e., low chance or high chance of OS. In addition to the chance of OS prediction, local interpretable model-agnostic explanations (LIME) and Shapley Additive exPlanations (SHAP) techniques were used to provide local (LIME) or both local and global explanations (SHAP) of the contributions of each variable to the predictive performance of the model. Detailed explanations of the LIME and SHAP techniques are provided in "Local interpretable model-agnostic explanations (LIME)" and "Shapley additive exPlanations (SHAP)", respectively.

### Local interpretable model-agnostic explanations (LIME)

LIME, the acronym for local interpretable model-agnostic explanations^26^, is a model agnostic technique that is applied to an already trained model to investigate and analyze the relationship between the input parameters and output represented by the model^27^. It is a local model interpretability technique that works by tweaking the input parameters while observing the effect of this tweak on the output^28^. The significance of the tweaking helps to understand the degree of accuracy of the prediction made by the model and to investigate which of the input variables caused the prediction of a data sample.

Technically, the LIME technique generates a new dataset which consists of perturbed samples and corresponding predictions from the initially trained model. Subsequently, an interpretable model is trained by LIME on the newly generated dataset by weighting the proximity of the sampled instances to the instance of interest (i.e., the training data’s mass center). This helps to achieve a good approximation of the model predictions locally, that is, for a single instance of prediction instead of the entire dataset. The LIME technique differs from other model interpretability techniques in the sense that it considers interpretability from each sample data point in contrast to others that consider it from the entire dataset. Hence, LIME provides local interpretability. This kind of approximation (accuracy) is known as local fidelity. Hence, LIME experiences a tradeoff between model fidelity and complexity. The constraint for LIME technique is given as:

1 $${LIME}_{explanation} \left(x\right)= \underset{\mathit{ }g\in G}{\mathrm{arg}\mathit{min}}L \left(f, g, {\pi }_{x}\right)+ {\Omega }_{(g)},$$

where ${LIME}_{explanation} \left(x\right)$ is the LIME explanation for an instance $\left(x\right). f$ is the Global model (Gradient boosting decision tree in this study). $f$ is the ${\mathbb{R}}^{d}\to {\mathbb{R}}.g$ is the Local surrogate model. *G* is the family or array of possible explanations. That is, class of potentially interpretable models. $g\in G$ is the An explanation considered as a model. *L* is the Loss measures the closeness of the explanation to the prediction of the global/original model. ${\pi }_{x }\left(z\right)$ is the Proximity measure of an instance *z* from *x*. That is, proximity which measures how large the neighborhood around instance $(x)$ using exponential smoothing kernel. ${\Omega }_{\left(g\right)}$ A measure of the complexity of the explanation $g\in G.$ That is, model complexity, e.g., the number of input features (the fewer, the better).

Based on the constraint formula (1), the principle of operation of LIME involves minimizing the *L* without making any assumption regarding global model, *f* (since LIME is model agnostic). The loss (*L*) is the measure of unfaithfulness of the local surrogate model (*g*) is in approximating the global model (*f*) in the locality defined by π(x).

### Shapley Additive exPlanations (SHAP)

SHAP is an acronym for Shapley Additive exPlanations, which was introduced by Lundberg and Lee in 2017^29^. It uses the principle of game theory to make local explanations of model’s predictions^29^. In the context of game theory, the model is considered the rules of the game while the input features are the potential players that may either participate in the game (observed feature) or not (feature cannot be observed). Therefore, the SHAP technique computes the Shapley values by evaluating the model under several different combinations of input features and calculating the average difference in the output (prediction) when a feature is present compared to when it is absent^30^. This difference is known as the Shapley value and represents the contribution of the feature to the prediction made by the model^30^. Hence, the Shapley values quantify the contribution of each feature to the prediction of a model for a given input^28,30^.

Technically, the SHAP technique returns Shapley values which express model predictions as linear combinations of binary variables that describe whether each covariate is present in the model or not^27^. Intuitively, it approximates each prediction $f\left(x\right)$ with $g ({x}^{^{\prime}})$, where a linear function of the binary variables (${z}^{^{\prime}} \in {\left\{0, 1\right\}}^{M})$ as in classification problem and of the quantities ${\varnothing }_{i}\in {\mathbb{R}}$ is defined by the *additive feature attribution methods* given in (2)^27^:

2 $$g\left({z}^{^{\prime}}\right)= {\varnothing }_{0}+ \sum_{i=1}^{M}{\varnothing }_{i}{z}_{i}^{^{\prime}},$$

The additive feature attribution method should satisfy the essential properties of *local accuracy*, *missingness*, and *consistency* for it to present a meaningful explanation of a single prediction. Hence, the additive method that satisfies these properties is given as:

3 $${\varnothing }_{i}\left(f,x\right)= \sum_{{z}^{^{\prime}}\subseteq { x}^{^{\prime}}}\frac{\left|{z}^{^{\prime}}\right|!\left(M-\left|{z}^{^{\prime}}\right|-1\right)!}{M!} [{f}_{x }\left({z}^{^{\prime}}\right)- {f}_{x} \left({z}^{^{\prime}}\backslash i\right)],$$

where $f$ is the Original prediction model to be explained, $g$ is the Explanation model, $x$ is the Available variables. ${x}^{^{\prime}}$ is the Selected variables, *M* is the Number of simplified input features, ${f}_{x }\left({z}^{^{\prime}}\right)- {f}_{x} ({z}^{^{\prime}}\backslash i)$ is the Quantity that expresses for each single prediction, the deviation of Shapley values from their mean: the contribution of the $i$-th variable.

Therefore, the SHAP technique uses Shapley values as an explanatory model that locally approximates the original model, for a given variable value $x$ (*local accuracy*) such that whenever a variable is equal to zero (Shapley value, that is, *missingness*). Similarly, if the contribution of a variable is higher in a different model, its corresponding Shapley value is also higher (*consistency*)^27^.

### Interpretability and explainability with LIME and SHAP techniques

For interpretability and explainability with LIME, we used the *LimeTabularExplainer* in *Python version 3.10.*0 to fit the training data of the global model (extreme gradient boosting [XGBoost] in this study). This generates a new dataset consisting of permuted samples of the training data and the corresponding predictions from the global model. LIME then trains an interpretable model (local surrogate model) based on the perturbed data generated from the original training data, which is weighted by the proximity of the sampled instances to the instance of interest. The learned model (i.e., local surrogate model) should be a good approximation of the model predictions locally (*local fidelity*), without necessarily being a good global approximation. Therefore, the interpretable and explainable model for an instance *x* (8th instance in this study) is the local surrogate model (*g* in 1) that minimizes the loss function (*L* in 1). It measures the closeness of the explanation to the prediction of the global model in the presence of possible explanations (*G* in 1) while the model complexity ${\Omega }_{\left(g\right)}$ is kept low (e.g., prefer fewer features). In this study, we used all the input features since we had fewer input features (n = 11). Thus, we examined the explanations of the contributions of each prognostic parameter to the predicted output of a particular predictive instance (8th instance in the training data) (Fig. 4).

Similarly, the SHAP technique computes the contributions of each feature to the final prediction of a decision of our XGBoost model (i.e., tree-based model) for any instance ${x}_{i}$. Specifically, it uses TreeSHAP to estimate the Shapley values of features in the model. These Shapley values provide a way to quantify the contribution of each feature to the prediction made by the model (Fig. 5). The Shapley values are computed by starting with a null model without any independent variables and then computing the average marginal contribution as each variable is added to this model in a sequence, that is, averaged over all possible sequences. Additive attribution method (2) was used to calculate and approximate the SHAP values on the entire dataset (Fig. 5).

Hence, it based on how many training samples went down paths in the tree with a computational complexity of $O({TLD}^{2})$, where T is the number of trees, L is the maximum number of leaves in any tree and D the maximal depth of any tree. Thereby, explaining the raw predictions from the leaf nodes of the trees. That is, computing the effect of each feature at each node by recursively traversing the tree from the root node to a leaf node and computing the contribution of each feature at each split along the way. The contribution is then weighted by the number of training samples that pass through that split, and the Shapley value of the feature is estimated as the sum of the weighted contributions across all paths that include the feature. Similarly, the specific contribution of the input feature to a certain prediction was examined (Figs. 6, 7). Both the LIME and SHAP techniques were implemented on the XGBoost trained model.

### External validation, performance metrics, and feature importance

Due to the rarity of NPC, we have used a combination of temporal and geographic external validations. Temporal validation lies between internal and external validation^24^. We complemented the temporal validation with a complete independent geographic external validation. The temporal external validation was done using reserved cases (n = 547) that were used neither in the training nor in the testing of the model. To complement the process of temporal validation, we used a dataset (n = 60) collected from the Helsinki University Hospital (HUS) (Research permission no: Dnro THL/1197/5.05.00/2012) for geographic external validation (Table 2).

**Table 2:** Baseline demographic and tumor characteristics of cohorts for temporal and geographic external validations.

Variables (definition)	Temporal EV (SEER, United States) Total (n = 547)	Geographic EV (Helsinki University Hospital) Total (n = 60)
Race (ethnicity of the patient)		
White	339 (62.0%)	60 (100.0%)
Black	100 (18.3%)	0 (0.0%)
Others	108 (19.7%)	0 (0.0%)
Age at diagnosis (age of the patient at diagnosis)		
< 40 years old (young)	169 (30.9%)	6 (10.0%)
> = 40 years old (old)	378 (69.1%)	54 (90.0%)
Gender (biological sex)		
Male	390 (71.3%)	39 (65.0%)
Female	157 (28.7%)	21 (35.0%)
Marital status (marital status of the patient at the time of diagnosis of NPC)		
Single	233 (42.6%)	0 (0.0%)
Married	314 (57.4%)	60 (100.0%)
AJCC 7th edition, T-stage (2010–2015)		
AJCC T1	183 (33.5%)	20 (33.3%)
AJCC T2	108 (19.7%)	10 (16.7%)
AJCC T3	123 (22.5%)	15 (25.5%)
AJCC T4	133 (24.3%)	15 (25.5%)
AJCC 7th edition, N-stage (2010–2015)		
AJCC N0	177 (32.4%)	24 (40.0%)
AJCC N1	215 (39.3%)	12 (20.0%)
AJCC N2	154 (28.2%)	23 (38.3%)
AJCC N3	1 (0.1%)	1 (1.7%)
AJCC 7th edition, M-stage (2010–2015)		
AJCC M0; No distant metastasis	497 (90.6%)	59 (98.3%)
AJCC M1; Presence of distant metastasis	50 (9.1%)	1 (1.7%)
Grade		
The differentiation of cancer cell		
Grade I: Well differentiated	27 (4.9%)	2 (3.3%)
Grade II: Moderately differentiated	93 (17.0%)	16 (26.7%)
Grade III: Poorly differentiated	255 (46.6%)	41 (68.3%)
Grade IV: Undifferentiated	172 (31.4%)	1 (1.7%)
Surgical resection		
Indication of the performance of surgery		
No surgery performed	465 (85.0%)	60 (100.0%)
Surgery performed	82 (15.0%)	0 (100.0%)
Radiotherapy		
This describes whether the patient receives radiation or not		
No exposure to radiotherapy	413 (75.5%)	0 (0.0%)
Exposure to radiotherapy	134 (24.5%)	60 (100.0%)
Chemotherapy		
No chemotherapy administered	106 (19.4%)	19 (31.7%)
Chemotherapy was administered	441 (80.6%)	41 (68.3%)
Overall status		
Alive	305 (55.8%)	18 (30.0%)
Dead	242 (44.2%)	42 (70.0%)

The results from the geographic external validation give the gold standard performance of the model (Table 3). The performance of the external validation process was evaluated in terms of concordance index (c-index), Area under curve (AUC), sensitivity, specificity, positive predictive value (PPV), negative predictive value (NPV), F1-score, accuracy, and Mathew’s correlation coefficient (Table 3).

**Table 3:** External validation for generalizability (temporal validation = 547 cases; geographic external validation = 60 cases).

	Performance metrics	Temporal validation (SEER, n = 547)	Geographic external validation (HUS, n = 60)
Confusion matrix parameters	True positive	248	14
	False positive	21	4
	False negative	56	10
	True negative	186	32
Predictive value	PPV (precision)	0.93	0.78
	NPV	0.77	0.76
Rate	False positive rate	0.10	0.10
	False negative rate	0.17	0.22
Other metrics	Sensitivity (recall)	0.83	0.58
	Specificity	0.89	0.89
	F1 score	0.87	0.67
Accuracy	Accuracy	85.9%	76.7%
	Balanced accuracy	86.0%	73.5%
Correlation	Mathew’s correlation coefficient	0.71	0.50
AUC	ROC of AUC	0.85	0.76
C-index	Concordance index	0.87	0.74

## Results

### Data description

The study cohort for ML model development included 1094 patients with nasopharyngeal cancer; 756 males and 338 females in a male-to-female ratio of 2.2:1. The mean age at diagnosis was 55.1 (SD $\pm$ 15.1: range 7–85) and the median age was 55.0 years. With regard to the tumor stage, the AJCC 7th TNM staging scheme showed that 393 (35.9%) had stage T1, 206 (18.8%) stage T2, 222 (20.3%) patients had stage T3, and 273 (25.0%) stage T4. Likewise, for the nodal parameter, 242 (31.3%) were N0, 389 (35.6%) were N1, 361 (33.0%) were N2, and 2 (0.2%) were N3; while 1001 (91.5%) for M0 and 93 (8.5%) were M1. Regarding histologic grading, 34 (3.1%) tumors were well-differentiated, 148 (13.5%) were moderately differentiated, 440 (40.2%) were poorly differentiated, and 472 (43.1%) were undifferentiated. The follow-up time ranged from 0 to 107 months (mean 50.2; median 51.5; SD $\pm$ 30.9). Other important parameters such as ethnicity, 462 (42.2%) were of White origin, 89 (8.1%) were Black, and 543 (49.6%) were from other origins including American Indian/AK Native and Asian/Pacific Islander. Considering marital status, 697 (63.7%) were married while 338 (30.9%) were considered unmarried (single, divorced, widowed, or separated) at the time of diagnosis (Table 1). Beam radiotherapy was the most common type of radiation given in this series. The clinicopathologic characteristics are briefly summarized in Table 1**.**

The average age of the cohort for temporal form of external validation (n = 547) at diagnosis was 55.1 (median: 57; SD $\pm 14.8;$ range 9–85). The male-to-female ratio was 2.5:1 where 390 were males and 157 were females. In terms of ethnicity, 392 (62.0%) were of White origin, 100 (18.3%) were Black, and 108 (19.7%) were from other origins including American Indian/AK Native and Asian/Pacific Islander. Considering marital status, 314 (57.4%) were married while 233 (42.6%) were considered unmarried (single, divorced, widowed, or separated) at the time of diagnosis (Table 2). The AJCC TNM tumor staging showed that 183 (33.5%) had stage T1, 108 (19.7%) stage T2, 123 (22.5%) patients had stage T3, and 133 (24.3%) stage T4. Likewise, for the nodal parameter, 177 (32.4%) were N0, 215 (39.3%) were N1, 154 (28.2%) were N2, and 1 (0.2%) were N3; while 497 (90.6%) for M0 and 50 (9.1%) were M1. With regard to grading, 27 (4.9%) tumors were well-differentiated, 93 (17.0%) were moderately differentiated, 225 (46.6%) were poorly differentiated, and 172 (31.4%) were undifferentiated. The follow-up time ranged from 0 to 107 months (mean 43.8; median 43.0; SD $\pm$ 28.6).

The HUS cohort for geographic external validation development included 60 predominantly Caucasian patients with NPC. Of these 60 patients, 378 (69.1%) were older than 40 years with a mean age at diagnosis of 56.9 (median 57: SD $\pm$ 11.9: range 30–82). The male-to-female ratio was 1.9:1 where 39 (65.0%) patients were male and 21 (35.0%) were females. With regard to grading, 2 (3.3%) tumors were well-differentiated, 16 (26.7%) were moderately differentiated, 41 (68.3%) were poorly differentiated, and 1 (1.7%) was undifferentiated (Table 2). In terms of the AJCC TNM tumor staging, 20 (33.3%) had stage T1, 10 (16.7%) stage T2, and 15 (25.5%) each for stage T3 and T4 patients. Likewise, for the nodal parameter, 24 (40.0%) were N0, 12 (20.0%) were N1, 23 (38.3%) were N2, and 1 (1.7%) were N3; 59 (98.3%) were M0 and 1 (1.7%) were M1 (Table 2). The follow-up time ranged from 0 to 215 months (mean 64.1; median 49.0; SD $\pm$ 57.5). Surgery was not a preferred treatment option for the HUS cohort (Table 2). Hence, all the patients in this cohort received definitive (chemo)radiotherapy treatment.

### Performance metrics for the algorithms

**Figure 3:**
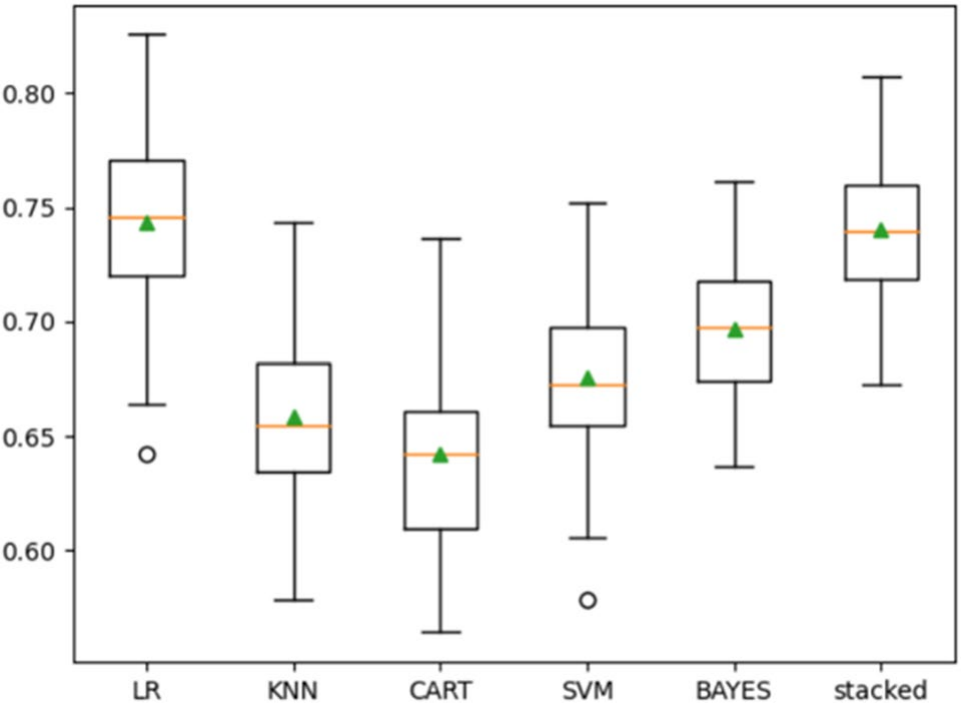
Training performance of the individual algorithm and the stacked algorithm.

The performance accuracy of the individual algorithms was 85.4%, 83.0%, 85.2%, 85.3%, and 85.9% for logistic regression, naïve Bayes, k-nearest neighbors, support vector machine, and decision tree algorithms (Fig. 3). When stacked together, a resulting accuracy of 85.9% was obtained for the stacked algorithm (Fig. 3). Therefore, the stacking of these algorithms did not show significant improvements in the accuracy of the model.

Similarly, the state-of-the-art XGBoost algorithm gave 84.5% performance accuracy. This indicates that XGBoost and the stacked algorithm examined in this study showed comparable performance. This result further demonstrated that the XGBoost may be capable of producing predictive performance that is comparable to five different individual algorithms combined. As a result, we performed a hybrid of temporal and geographic external validations on the XGBoost algorithm (Table 3). The performance metrics of the XGBoost with geographic external validation were reported as the gold standard performance in this study (Table 3).

### Temporal and geographic external validation performance metrics

The temporal external validation of XGBoost produced an accuracy of 85.9% and c-index of 0.87. Likewise, the performance accuracy of XGBoost was externally validated with a geographic cohort from HUS, producing an accuracy of 76.7% and c-index of 0.74. The specificity for both temporal and external geographic cohort was 0.89. In terms of precision (positive predictive value) and negative predictive value, the XGBoost showed a precision of 0.93 for temporal validation and 0.78 for external geographic validation. The negative predictive value was 0.77 for temporal validation and 0.76 for external geographic validation. Other performance metrics from the hybrid validation approach are given in Table 3.

### Explainability and Interpretability of the XGBoost model

**Figure 4:**
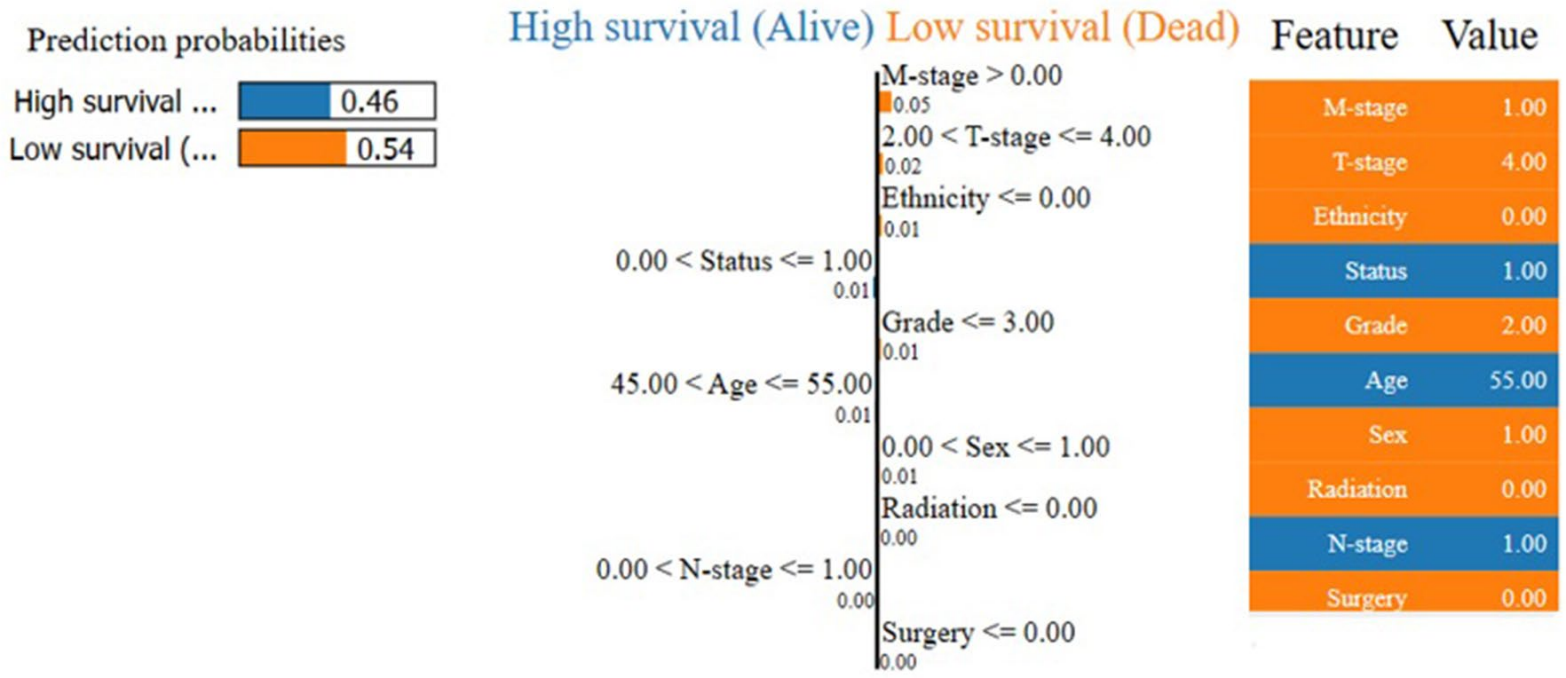
LIME explainability of a single instance.

The LIME technique further explains and interprets the prediction of an instance (i.e., for an individual patient) (Fig. 4).

For example, the outcome prediction of the patients shown in Fig. 4 indicates that this particular patient has low chance of OS due to NPC with 54.0% prediction confidence. In addition, it further explains the rationale for the prediction by indicating how the input features (e.g., T-stage = advanced-stage, M-stage = metastasized to other parts, tumor grade = poorly differentiated, ethnicity = white origin, and gender = male) have contributed to the predicted outcome (low chance of OS).

**Figure 5:**
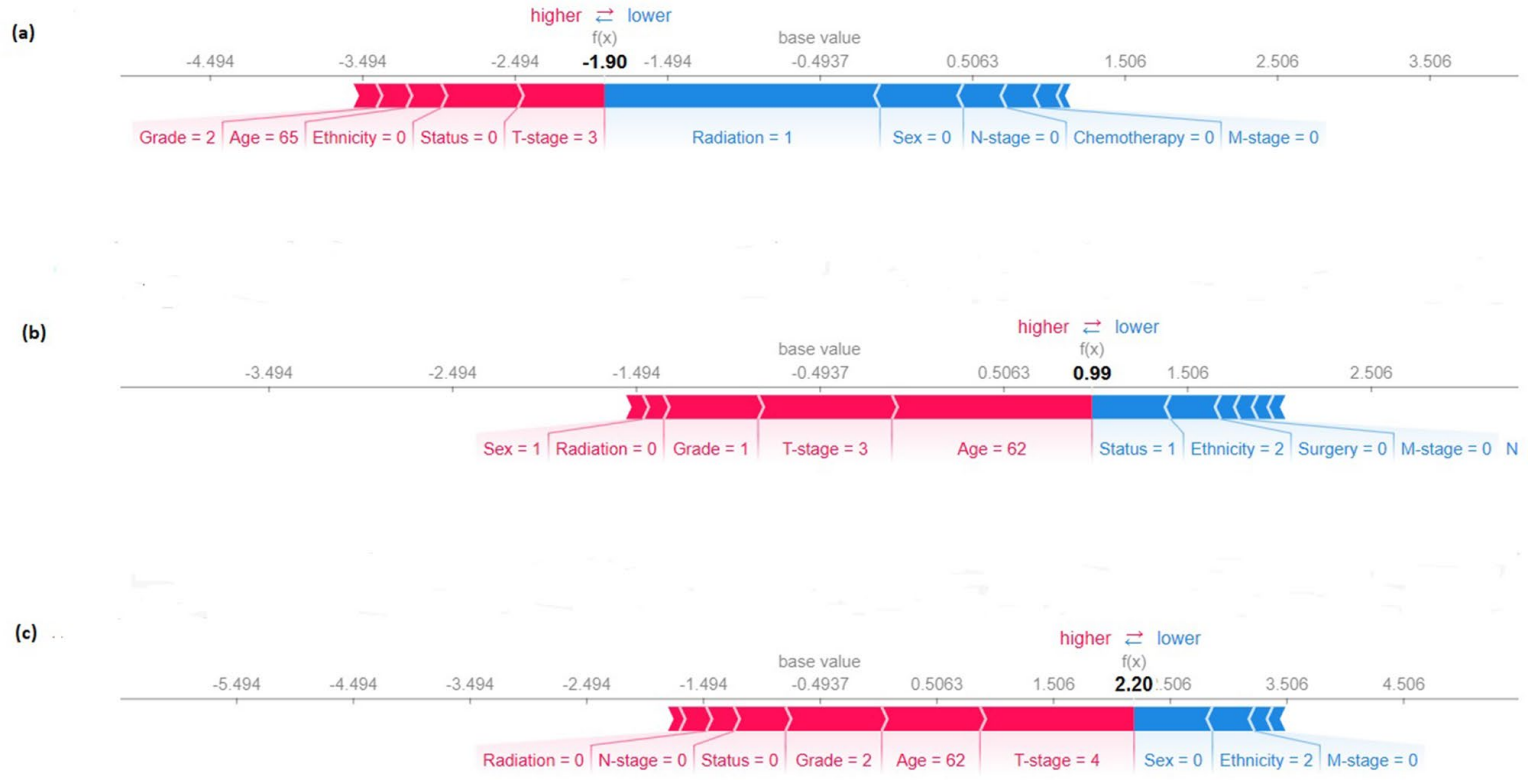
SHAP force plot showing (**a**) high chance of survival (**b**,**c**) low risk of survival.

**Figure 6:**
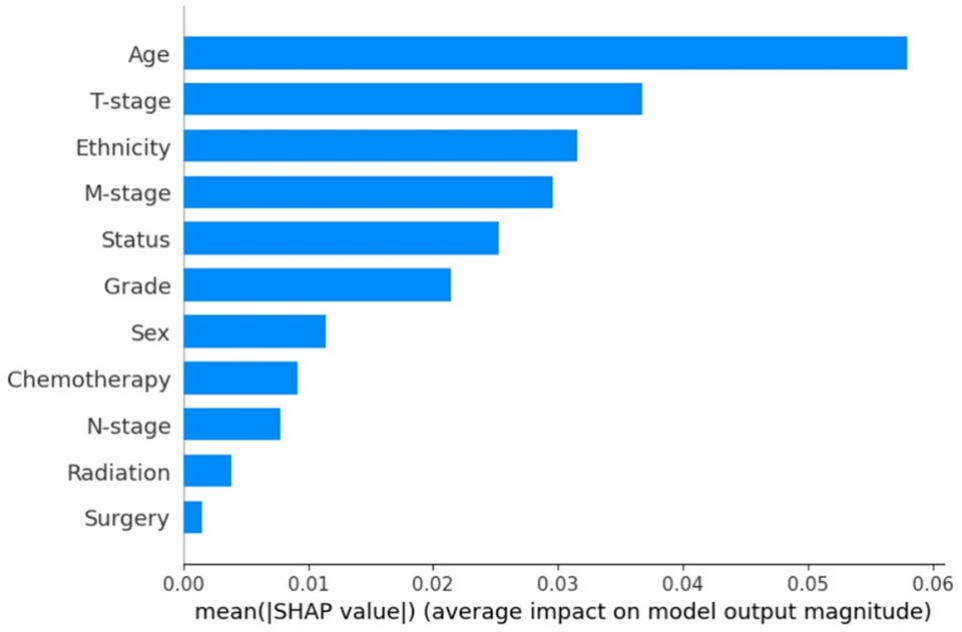
Overall contribution of each feature to the prediction.

Similarly, SHAP technique provides an explanation for the prediction of an outcome by computing the contribution of each feature to the prediction [local and global explanations] (Figs. 5, 6). From Fig. 5 (local prediction—individual predictions made by the model), the model’s predictive probability value $[f(x)]$ were − 1.90, 0.99, and 2.20 for NPC patients 1–3, respectively. As shown in Table 1, the label of the target outcome indicated that 0 means high chance of survival and 1 signifies low chance of survival. Therefore, the model’s predictive probability for the first patient was high chance of survival while low chance of survival was predicted for the second and third patients (Fig. 5a–c).

The numbers on the plot arrows are the value of the input feature for each patient (Fig. 5). The bigger the arrow, the bigger the impact of the feature on the output. Therefore, for the first patient with $\left[f\left(x\right)\right]= -1.90$, marital status (unmarried), grade (moderately differentiated), ethnicity (Caucasian), age of the patient (65 years), and T-stage (T3) have a negative contribution (Fig. 6a) to predicting the patient as having a high chance of survival while other input features have a positive contribution to the predicted outcome. Remarkably, both the marital status and T-stage have almost equal positive contributions since they both have equal arrow sizes while the age of the patient, grade and ethnicity also have equal contributions to the prediction made by the model since they have almost equal arrow sizes (Fig. 5).

Likewise, for the second patient with $\left[f\left(x\right)\right]= +0.99$, radiation (no radiation treatment), sex (male), grade (well differentiated), age of the patient (62 years), and T-stage (T3) have a positive contribution (Fig. 5b) to predicting the patient as having a low chance of survival while other input features have a negative contribution to the predicted outcome. Similarly, for the third patient with $\left[f\left(x\right)\right]= +2.20$, radiation (no radiotherapy given), N-stage (no distant metastasis), marital status (unmarried), grade (moderately differentiated), age of patient (62 years), and T-stage (T4) have a positive contribution (Fig. 5c) to predicting the patient as having a low chance of survival. The global contribution of each variable to the overall predictive ability of the model is presented in a SHAP beeswarm plot (sub "Explainability and interpretability of the XGBoost model").

### Evaluating the input variables for importance

**Figure 7:**
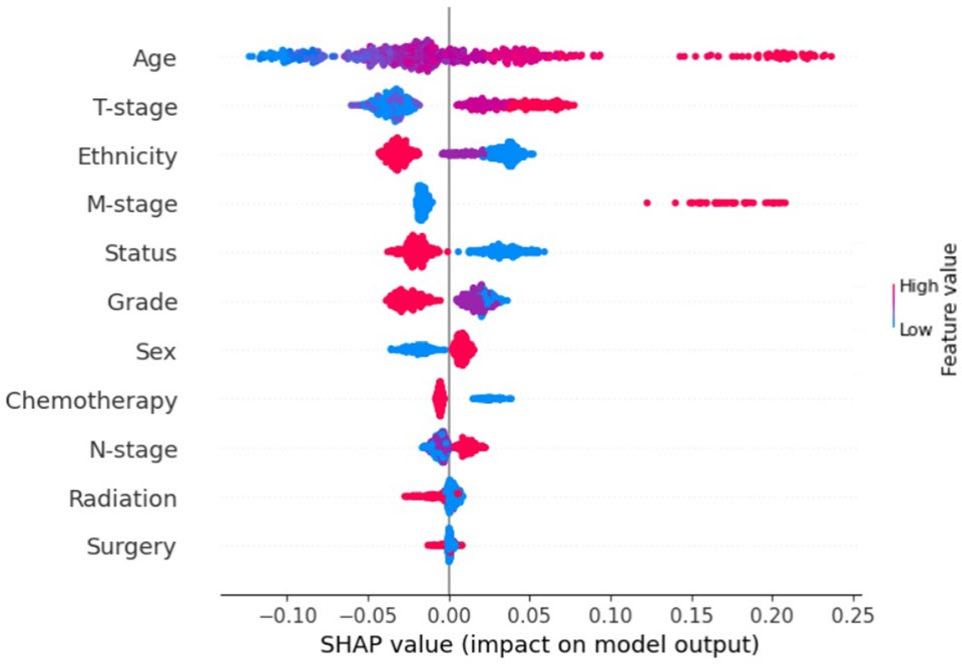
SHAP beeswarm summary plot on the impact of input variables on the XGBoost model’s predictive ability.

The feature importance of the input variables based on the SHAP technique showed that, in decreasing order of significance, the age of the patients, T-stage, ethnicity, M-stage, marital status, and grade of the tumor were found to be the input variables that had a significant influence on the model’s performance to predict the chance of OS in NPC patients (Fig. 6). Likewise, the detailed contribution of these variables to chance of survival of NPC is presented in Fig. 7.

The SHAP beeswarm plot further provides detailed explanations of how the parameters contained in each variable contribute to the outcome of interest (global explanation and interpretation).

As shown in Fig. 7, the expected outcome can either be a high chance of survival (negative side on the x-axis) or a low chance of survival (positive side on the x-axis). Therefore, the details of the effect of each of the prognostic parameters were presented in Fig. 7. Therefore, it was found that lower age, lower T and M (tumor and distant metastasis) stage, married, Asian ethnicity, gender (female), and non-surgical treatment (chemotherapy and radiotherapy) were associated with a higher chance of OS of NPC (Fig. 7).

## Discussion

We leveraged the promising predictive performance of five different individual algorithms by combining them into a single and unique algorithm (stacked algorithm) that has a high predictive performance accuracy. The stacked algorithm was used to develop a machine learning (ML) system for chance of survival prediction of nasopharyngeal cancer (NPC) patients. Considering the growing application of the extreme gradient boosting (XGBoost) algorithm in many clinical applications due to its outstanding performance, we compared the predictive performance of the stacked algorithm with another model developed using the XGBoost algorithm. The comparison was based on an independent geographic external validation cohort from the Helsinki University Hospital (HUS). We found out that both the stacked algorithm and the XGBoost algorithm performed comparably in the survival chance stratification of NPC patients.

Furthermore, owing to the continued criticism of ML models because their predictions are often untransparent and uninterpretable, we incorporated explainability and interpretability to the predictions made by the XGBoost model using LIME and SHAP techniques. These techniques highlight patient-specific information on how each variable contributed to the chance of OS predicted by the model (local interpretation), extent of accuracy of the predicted chance of OS (local explanation) for a particular patient, and how each of these variables contributed to the predicted performance of the model (global explanation and interpretation). This approach is geared towards personalized management of NPC cancer.

In the past, several ML algorithms have been employed in the prognostication of outcomes in various subsites of head and neck cancer^18,20,31–35^. However, there is a growing trend to explore the potential of ML in the evaluation of prognoses, specifically, in nasopharyngeal cancer^18,20^. For example, the study by Oei et al. specifically compared the ML approach with traditional statistics and found that ML outperformed these^20^. Similarly, the study by Akcay et al. compared various individual ML algorithms in the prognostication of outcomes in NPC patients^18^. However, these studies used a relatively small number of cases. In addition, the developed models were neither externally validated nor explainable. We focused on this research gap by exploring the potential of a stacked ML algorithm that combines five individual ML algorithms in the prognostication of OS in NPC using a relatively large number of cases. Based on the promising results obtained in this research field, various modifications were made to the underlying ML algorithms for improved performance. An example of these modifications is the effective implementation of the gradient boosting ensemble paradigm to achieve the extreme gradient boosting ML algorithm or XGBoost for short^36^. We thus examined the use of this powerful algorithm for prognostication of OS in NPC patients. Furthermore, this study leveraged the potential of LIME and SHAP techniques to provide explanations and interpretations of the predictions made by the model. Specifically, the SHAP technique is poised to further provide explanations on the significance of each input variable on the OS chance stratification performance of the XGBoost model.

Despite the combination of multiple algorithms to form the stacked model, the XGBoost model alone produced a comparable performance. This is because the algorithm has been built to generate a series of iteratively constructed tree models where the trees are added one at a time to the ensemble and fit so that the prediction errors made by the prior models are adequately corrected^36,37^. This architecture (boosting approach) enhances model performance^36,37^. Hence, it is a computationally efficient (i.e. fast to execute) ML algorithm that is based on a scalable end-to-end tree boosting system architecture^36^. Considering the continued proliferation of medical data and the quest for personalized and precision medicine, the extreme gradient boosting algorithm offers the potential to be the ML of choice as it is able to provide remarkably fast execution speed and model performance^36^.

The predictive accuracy shown by the trained ML model is posited to provide an accurate, objective, and lower cost assistive tool to the clinicians^18^. This model may provide an improved opinion to the clinicians to complement the TNM staging system in survival prognostication by incorporating multiple parameters. Such a strategy is important to provide individualized treatment planning for NPC patients. Besides the predictive performance of the XGBoost model, both the Local Interpretable Model Agnostic Explanations (LIME) and SHapley Additive exPlanations (SHAP) techniques provide the rationale for the predicted outcomes by the model.

The LIME and SHAP techniques are both model-agnostic techniques for providing explanations to the prediction made by an ML model^27,29^. These techniques can interpret the complex relationships between the input features and the target outcome. For example, the LIME approach reveals the degree of probability of correctness of the prediction and how each factor has contributed to the possible outcomes (Fig. 4). This extra functionality provided by the LIME technique is posited to provide a transparent ML model, especially, regarding the predicted outcome. Consequently, clinicians as decision-makers and other stakeholders have greater visibility, understanding, and trust regarding the explanations of the decisions that lead to the model’s output. The SHAP technique, on the other hand, provides explainability and interpretability as provided by LIME but in a more detailed and compact manner using the game’s theoretically optimal Shapley values.

The main difference between LIME and SHAP techniques is that LIME only provides an explanation and interpretation for a single prediction made by the ML model (local interpretation) while SHAP provides the contribution of each input variable to the prediction made by the model (local explanations and interpretations) (Figs. 5, 7). Additionally, the SHAP technique examines the contribution of each input variable to the overall predictive ability of the model (global explanations and interpretations) (Fig. 6). Unlike the traditional feature importance that provides the general overview of the input variables, the SHAP-based feature importance further reveals how the parameters contained in each variable have contributed to the overall predictive capability of the model (Fig. 5).

The significance of the input variables to the OS prediction using SHAP technique showed that age, T-stage, ethnicity, M-stage, marital status, and grade were among the most important prognostic factors, in decreasing order of significance (Fig. 6). Specifically, lower age, lower T and M (tumor and distant metastasis) stage, married, Asian ethnicity, gender (female), and non-surgical treatment (chemotherapy and radiotherapy) were associated with a higher chance of OS of NPC (Fig. 7). This observation has been corroborated and highlighted by several studies^12,38,39^, for example, the study by Zhu et al., demonstrating the prognostic role of age in a series of 469 NPC patients^12^. Occurrence of NPC increases steadily with age and the peak incidence occurs at different ages but usually between the ages of 40–59 years^12,40–42^. Therefore, efforts should be made by various organizations to define the threshold to stratify the patients as either young or old NPC patients, and to provide treatment guidelines for both groups^18^. Owing to the identification of age by the evaluated ML model as an important factor in this study, it is of great importance to recognize age-specific differences in NPC in terms of targeted treatment modalities^12^.

Our ML model identified ethnicity as one of the important factors for survival. This is corroborated by the fact that NPC is endemic in Southern China and Southeast Asia^43–45^. Distant metastasis was deemed important by our SHAP technique for the prognostication of OS in NPC patients. It has been reported to be the most important negative prognostic factor in nasopharyngeal cancer^46–48^, as advanced distant metastasis was associated with significantly poorer disease-free survival and OS^44,49,50^. This result was supported by other studies indicating that recurrence and distant metastasis are presently the main reasons for suboptimal treatment outcomes in NPC^51,52^. In addition, as NPC originates close to an area with abundant lymphatic network, it has a higher tendency to metastasize than carcinomas from other subsites of the head and neck^10,53^.

Notably, the prognosis of patients with distant metastasis at diagnosis differs from the patients who developed distant metastasis after treatment^54–59^. At the same time, the AJCC staging scheme does not seem to provide an insightful solution to the above-mentioned difference in prognosis of patients with distant metastasis since the staging scheme considers patients with distant metastasis as a single group^54,60,61^. Therefore, the application of the ML model as a chance of survival stratification system that enables prediction of OS in NPC patients even at diagnosis is valuable for both therapeutic decision-making and research.

Radiotherapy constitutes the treatment of choice for NPC^46–48,54,62,63^ and systemic chemotherapy remains an important adjuvant modality^54^. However, neither of these primary treatment modalities was highlighted by the SHAP technique as being among the most significant in prediction of the OS in NPC. Instead, a combination treatment strategy involving radiotherapy and chemotherapy appears most useful for OS in NPC^64,65^. The use of intensity-modulated radiotherapy offers an effective treatment approach to improve OS rates in NPC^66–68^.

In conclusion, the use of disruptive technologies such as the ML algorithms to estimate the prognosis of NPC is poised to address the disparities in the AJCC TNM staging scheme and heterogeneous treatment outcomes in NPC patients with distant metastasis. This is because the ML approach can provide accurate prognostication for the outcome in targeted treatment planning. The ML paradigm can examine the complex relationships between these variables. Furthermore, it is capable of analyzing how similar patients have responded in the past, and thereby also predicting the outcome of the new patient under consideration. The prognostication of outcome in this complex and heterogeneous group of diseases is important in guiding treatment planning accordingly. Therefore, combining highly accurate and state-of-art ML models with explainability and interpretability provides a promising way for survival chance stratification of NPC patients.

Our study has some limitations. First, our model was developed using data obtained from the SEER database, which is one of the largest publicly available cancer databases for the United States population. Thus, the data obtained from this database are not directly applicable to the EBV-associated endemic NPC seen in Southern China and Southeast Asia. Remarkably, the EBV variant in NPC belongs to WHO classification type 3, versus the other types seen in sporadic NPC. Therefore, the proposed model in this study cannot be immediately related to an Asian NPC dataset because of the inherent differences in the NPC. Second, the ML model was developed based on retrospective data. Third, the developed model was validated externally with a hybrid of temporal and geographic external validation paradigms. Further geographic external validation is warranted with a relatively large number of cases. The overall performed metrics of the model showed that the model may be retrained with a more balanced dataset. In spite of these shortcomings, the ML model still showed a significant predictive ability. Further validation in other populations, including Asian patient groups is important and prospective studies are warranted. Relating to Asian patient groups, it has been published that integrating the plasma EBV-DNA into the TNM staging will further discriminates the prognostic implications. Therefore, in future studies, AI models can be developed using such data. Such models may serve as ancillary tools for chance of survival stratification and management guidance. The model performance can be improved through federated learning in the future.

## Data Availability

The datasets generated from the current study are available from the corresponding author on reasonable request.
